# Developing perennial wildflower strips for use in Mediterranean orchard systems

**DOI:** 10.1002/ece3.10285

**Published:** 2023-07-17

**Authors:** Alice Mockford, Alberto Urbaneja, Kate Ashbrook, Duncan B. Westbury

**Affiliations:** ^1^ Department of Landscape Ecology, Institute for Natural Resource Conservation Kiel University Kiel Germany; ^2^ School of Science and the Environment University of Worcester Worcester UK; ^3^ Instituto Valenciano de Investigaciones Agrarias (IVIA) Centro de Protección Vegetal y Biotecnología Moncada Spain

**Keywords:** *Citrus*, ecosystem services, habitat management, mowing, orange orchards, plant provided resource

## Abstract

To support sustainable food production and the delivery of ecosystem services through ecological intensification, wildflower strips have become a popular strategy. Despite their success in temperate orchard systems, they remain understudied in Mediterranean ecosystems, which poses a significant barrier to uptake. In order to further promote their adoption, seed mixes must be optimised for commercial orchard systems and for the Mediterranean climate. Plant species should be selected for their consistent performance, whilst the availability of resources for ecosystem service providers determines the quality of the wildflower strip. In this study, the suitability of 12 native perennial forbs and two tussock‐forming grass species for wildflower strips in commercial *Citrus* orchards was assessed over a 3‐year period. Distinct resources for natural enemies according to the different plant growth stages were used an indicator of wildflower strip quality. The wildflower strips were managed under two different cutting strategies: (i) standard management, in which wildflower strips were cut once annually in February, and (ii) active management, in which wildflower strips were cut two additional times each year. The establishment and success of the sown species were compared. The influence of wildflower strips and their management on plant species richness, community structure, and the provision of resources was compared with a control treatment, in which alleyways were managed conventionally by cutting any naturally occurring vegetation to a height of ≤5 cm, four to five times annual. For the first time, the performance of native perennial plant species has been assessed in Mediterranean orchard systems and a seed mix developed for targeting pest regulation services. The wildflower strips were successful in increasing plant species richness and the available resources expected to support natural enemies. However, only wildflower strips managed with cutting once annually enhanced vegetation cover relative to the control, whilst extending the flowering period. This study therefore provides crucial tools for the further development of sustainable approaches to food production in Mediterranean orchard systems.

## Graphical abstract

For the first time, native perennial wildflower strips were established in Mediterranean *Citrus* orchards, which increased plant species richness, supported distinct plant communities, and boosted resources expected to support natural enemies. Eight of the sown forb species and one of the tussock‐forming grass species are recommended, and wildflower strips should be managed by cutting once annually. The study shows great potential for perennial wildflower strips to be used in other Mediterranean cropping systems where there is a real need to support sustainable production.

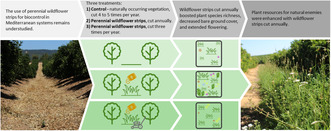

## 1 INTRODUCTION

Facing the increased degradation of natural habitats, the loss of biodiversity is impacting ecosystem function (Cardinale et al., 2012). Pressure is therefore mounting to identify sustainable solutions to agriculture (Cassman & Grassini, 2020). The use of wildflower strips to support the biodiversity which underpins food production has become a popular approach (Haaland et al., 2011). By establishing farm‐scale ecological infrastructure, wildflower strips can support beneficial arthropods and the supply of ecosystem services (Sutter et al., 2018). Such approaches to sustainable production have been successful in a variety of temperate orchard crops, including cherry, apple and blueberry (Albrecht et al., 2020). However, the performance of native perennial plant species in wildflower strips has not been investigated in Mediterranean orchards systems.

There is strong rationale to use native perennial plant species in wildflower strips rather than non‐native and annual or biennial species (Isaacs et al., 2009). Non‐native herbaceous species tend to harbour greater proportions of potential pest species than native vegetation and can facilitate the early colonisation of pest species on the crop (Parry et al., 2015). Additionally, the quantity of floral resource provided by annual and biennial plant species varies significantly between years (Campbell et al., 2017) and the regular cultivation to maintain them contributes to soil erosion and carbon release (Rosa‐Schleich et al., 2019). In contrast, native perennial species are adapted to the regional climate, typically require lower water and nutrient inputs, and do not require annual sowing (Frank et al., 2008). This therefore presents a more sustainable and economically viable solution for growers (Miettinen et al., 2014). For beneficial arthropods, year‐on‐year resource is more consistent in perennial swards (Carvell et al., 2006), which becomes more resource rich with continued development and typically exhibit longer flowering periods (Fiedler & Landis, 2007). Thus, native vegetation can support a greater abundance and diversity of beneficial arthropods than non‐native vegetation (Tuell et al., 2008) and a greater natural enemy‐to‐pest‐ratio than the crop (Bianchi et al., 2013; Parry et al., 2015). Furthermore, increasing plant species richness can support a greater abundance of natural enemies (Marshall & Moonen, 2002). Additionally, plant species richness tends to also increase plant trait diversity, which is a key driver of natural enemy diversity (Woodcock et al., 2007, 2009). Such habitats can then serve as sources for beneficial arthropods to spill over onto the crop (Bianchi et al., 2013; Parry et al., 2015).

Wildflower strips must be managed by cutting not only to maintain plant communities, but also to allow access for crop management (Bugg & Waddington, 1994). However, the number and timing of cuts can affect plant community composition and structure (Westbury et al., 2008), resource availability (Mockford et al., 2022) and the assembly of higher tropic levels (Woodcock et al., 2009). Late spring cutting selects for perennial species by promoting vegetative shoot growth and preventing annuals from setting seed (Marriott et al., 2003; Westbury et al., 2008). Cutting can also be used as a strategy to manipulate the availability of required resources, for example shelter, carbohydrate and protein throughout the year (Bugg & Waddington, 1994). Cutting prior to bud burst, for example, may extend the flowering period (Nowakowski & Pywell, 2016). In temperate climates, cutting is typically recommended once annually in late summer to 15 cm, to support nectar‐feeding arthropods (Natural England, 2013); however, the management applied to Mediterranean native perennial wildflower strips has not yet been investigated.

*Citrus* is an important global cash crop traded all over the world, with annual total production of 143.8 million tonnes (FAO, 2021). Spain is considered the leading global exporter (FAO, 2021). In the Mediterranean basin, Spain comprises the largest land use area for citriculture (Berk, 2016), totalling 269,441 ha in 2021 (MAPA, 2022), of this, 49% is dedicated to orange production (MAPA, 2022). Most of the sweet orange, such as Navel, grown in Spain is destined for the high‐value fresh fruit market (FAO, 2021) for which high quality and high aesthetic standards are expected (Urbaneja et al., 2020). Consumer demand for products low in pesticides residuals and organically produced has increased (Shafie & Rennie, 2012). Therefore, there is strong rationale to support ecological intensification and enhance ecosystem service delivery in orange production. As such, commercial Navel orange (*Citrus sinensis*) orchards were used in this study.

*Citrus* orchards support a great richness of native and naturalised natural enemies (see Jacas & Urbaneja, 2010), but key pests still escape satisfactory management below economic injury levels (Urbaneja et al., 2020). Consequently, there is much justification to support natural enemies in orchards and harness their pest regulation services. Typically, alleyways between rows of *Citrus* trees are maintained as bare soil by treating naturally occurring vegetation with herbicide, cultivation or regular cutting (Monzó et al., 2020). Such methods negatively impact the richness and abundance of natural enemies and limit pest regulation services (Aguilar‐Fenollosa, Ibáñez‐Gual, et al., 2011; Gómez‐Marco et al., 2016). In Spain, it is becoming increasingly common for growers to leave naturally occurring (unsown) vegetation in alleyways between rows of fruit trees to limit soil erosion, which is managed with regular cutting (Jacas & Urbaneja, 2010). Previous attempts to support natural enemies by sowing seed mixes in Mediterranean *Citrus* orchards have failed (Silva et al., 2010), possibly due to the selection of a limited number of plant species, which possess a limited variety of traits, coupled with the use of agricultural varieties. This highlights the importance of plant selection and subsequent management strategies which are based on plant species performance and the successful establishment of plant communities which provide resources for natural enemies.

To maximise benefits to natural enemies, plant species composition requires careful consideration (Duru et al., 2015). Wildflower strips must provide key resources that increase natural enemy longevity and fecundity, such as carbohydrate (nectar and honeydew), shelter and refuge and protein (pollen and alternative hosts/prey) (Gurr et al., 2017). Vegetation height and structure are key drivers of arthropod assemblages, with more complex swards promoting larger and more predatory species (Woodcock et al., 2007, 2009). Vegetation structural heterogeneity is closely related to plant species and trait diversity (Woodcock et al., 2009). Grasses forming dense tussocks, such as Orchard Grass (*Dactylis glomerata* L. (Poales: Poaceae)) and Tall Fescue (*Schedonorus (Festuca) arundinaceus* L. (Poales: Poaceae)), provide microclimate shelter (Luff, 1965), and sustain alternative prey and hosts (Gómez‐Marco et al., 2016) for natural enemies (Thomas et al., 1991). Grass strips sown with *S. arundinaceus* have been adopted by some *Citrus* growers to help manage spider mites and thrips (Aguilar‐Fenollosa, Ibáñez‐Gual, et al., 2011; Jacas & Aguilar‐Fenollosa, 2013). Unsown forbs which establish in these otherwise species‐poor habitats further enhance the management of aphids (Gómez‐Marco et al., 2016), most likely due to the increased provision of pollen for coccinellids and nectar for parasitoids. However, responses are site‐specific due to the variability in plant species presence between sites. Selecting flowers with exposed nectaries provides accessible carbohydrate for natural enemies (van Rijn & Wäckers, 2016), increasing their abundance (Campbell et al., 2012). By diversifying forb species to provide floral resources in succession, natural enemies are supported throughout the year (Mockford et al., 2022). Due to a lack of studies, there are few candidate plant species for perennial Mediterranean wildflower strips and no data pertaining to their performance in commercial orchards, which represents a key barrier to uptake for growers (Girling et al., 2022).

The main objective of this study was to develop the use of perennial wildflower strips in a Mediterranean orchard system using a novel seed mix designed to provide resources for natural enemies. Three alleyway management approaches between rows of orange trees were investigated; (i) a control treatment for which alleyways were managed conventionally by cutting the naturally occurring vegetation to a height of ≤5 cm, four to five times annually, (ii) wildflower strips established in alternative alleyways managed by cutting once annually in February (hereafter SMWT) and (iii) the same sown wildflower strips as in the SMWT, managed actively by cutting two additional times per year (≈10 cm) in May and July (hereafter AMWT). The aims of this study were to investigate; (i) the performance and success of the sown species, (ii) the influence of alleyway management on plant species richness and composition and (iii) resource availability.

## 2 MATERIALS AND METHODS

### 2.1 Site description

**FIGURE A1:**
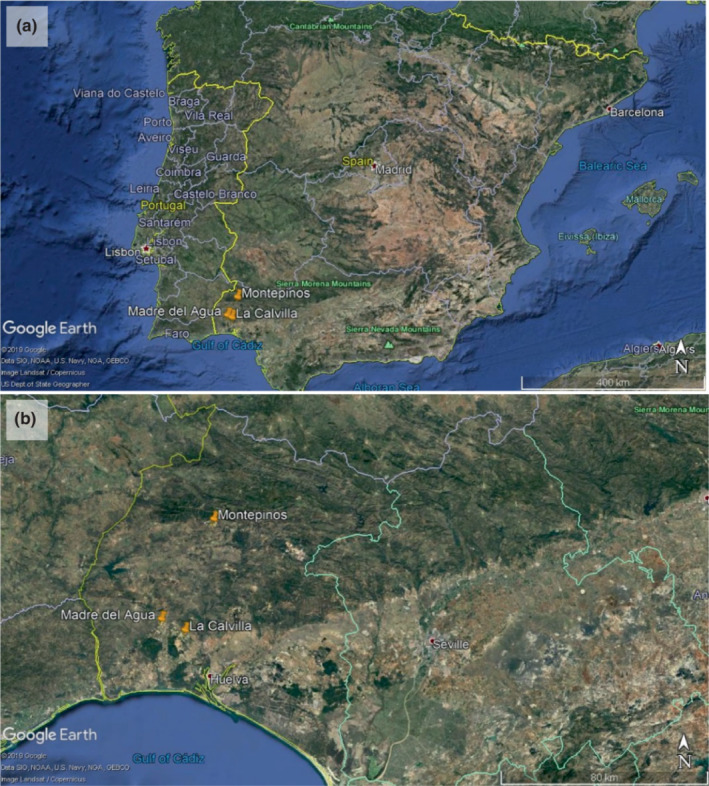
Location of the three orchards in relation to (a) the Iberian Peninsula and (b) the province of Huelva, south‐west Spain.

**FIGURE A2:**
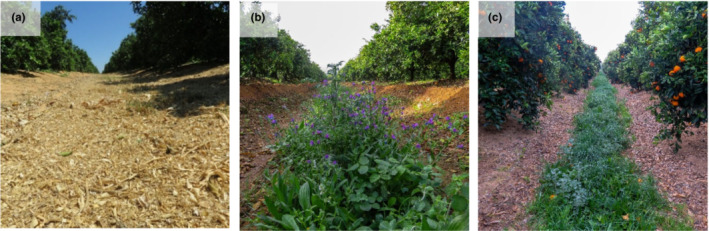
Treatment plots; (a) control treatment, managed conventionally by cutting throughout the season to keep the vegetation low; (b) standard management wildflower treatment, cut once annually, allowed to grow tall and complex; (c) active management wildflower treatment, cut three times annually to approximately 10 cm to encourage spillover of natural enemies onto the crop.

The 3‐year study was conducted in three commercial navel orange (*Citrus sinensis*) orchards in Huelva, Andalusia (Appendix A: Figure A1): Madre del Agua (37°26′27.80″N 7°9′55.73″W) and La Calvilla (37°24′10.95″N 7°3′42.67″W) in southern Huelva and Montepinos (37°47′43.21″N 6°56′21.11″W) in northern Huelva. All orchards were managed under Integrated Pest Management (IPM) guidelines (Llorens Climet & Martín Gil, 2014) and contained naturally occurring vegetation in alleyways, managed with regular cutting to a height of ≤5 cm, four to five times annually (Figure A2).

### 2.2 Study design

**FIGURE 1:**
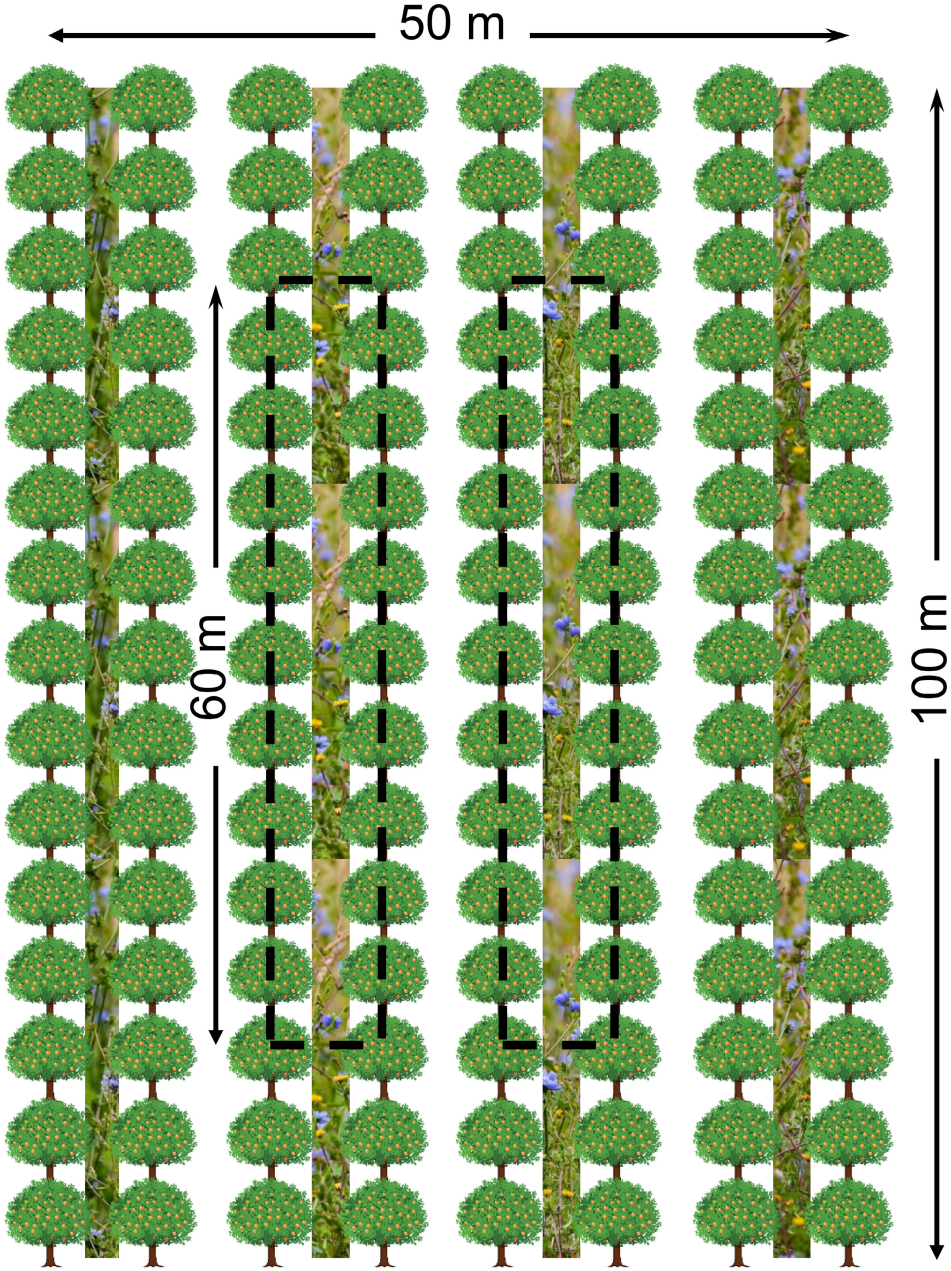
Schematic diagram of a 0.5 ha experimental plot, consisting of eight rows of orange trees and seven alleyways. Wildflower strips were established in alternate alleyways. Black dashed lines delimit the surveying areas.

A randomised block design was established with four complete replicate blocks in November 2016. The experimental treatments were applied in 0.5 ha plots, separated by at least 150 m. Each plot measured 100 m in length and 50 m wide and consisted of eight rows of orange trees and seven alleyways between rows (Figure 1). The wildflower strips were sown in November to take advantage of seasonal rainfall and the associated increase in germination success (Ramírez & Lasheras, 2015). Competition from existing vegetation was eliminated by applying glyphosate (RoundUp, Monsanto and Missouri) (Natural England, 2013). After 7 days, the alleyways were then cultivated to create a fine seedbed (Westbury et al., 2017). The seeds were mixed with sand to ensure even sowing by hand, immediately after which the seedbed was rolled to firm the seeds with the substrate. The novel seed mix consisted of all native perennial species, including 12 forb and two tussock‐forming grass species. Species were selected to provide floral resource in succession across the length of the year and a diversity of plant traits (such as phenology, growth forms, height and floral traits) to increase sward structure, provide a diversity of plant growth stages and support natural enemies (Table 1). The seed mix was sown in alternate alleyways at a rate of 5.66 gm^−2^ to create wildflower strips measuring 2 m wide and 100 m long (Table 1). During the establishment year of 2017 (Year 1), all wildflower plots were managed by cutting once in March and once in April to ≈10 cm to promote the establishment of the sown species (Woodcock et al., 2009).

**TABLE 1:** Species flowering periods and sowing rates (expressed as the percentage weight of seeds sown and actual seed number per species per square metre). Seeds were sown at a total rate of 5.66 gm^−2^.

Scientific name	Family	Flowering period	Sowing rate (%)	Actual sowing rate (g ha^−1^)
*Anchusa azurea*	Boraginaceae	March–June	1.12	63.39
*Salvia verbenaca*	Lamiaceae	January–May	1.53	86.60
*Psoralea bituminosa*	Fabaceae	April–July	2.47	139.80
*Hypericum perforatum*	Hypericaceae	May–August	6.11	345.83
*Mentha suaveolens*	Lamiaceae	June–August	7.35	416.01
*Ononis natrix*	Fabaceae	April–July	8.23	465.82
*Plantago lanceolate*	Plantaginaceae	March–September	8.23	465.82
*Dactylis glomerata*	Poaceae	May	8.29	469.21
*Schedonorus arundinaceus*	Poaceae	May–June	8.29	469.21
*Helichrysum stoechas*	Asteraceae	April–July	8.29	469.21
*Marrubium vulgare*	Lamiaceae	February–August	8.29	469.21
*Achillea millefolium*	Asteraceae	May–July	8.58	485.63
*Tanacetum vulgare*	Asteraceae	July–September	8.58	485.63
*Cichorium intybus*	Asteraceae	May–July	14.64	828.62
Total			100.00	5660.00

To reduce edge effects, the outermost alleyways within the 0.5 ha plots were excluded from sampling and a 20 m buffer region was established at either end of the alleyways. As such, a 60 m‐long central sampling area consisting of two alleyways between four rows of orange trees was established (Figure 1) (Englund & Cooper, 2003).

### 2.3 Botanical surveys

Botanical surveys were conducted in May of each year to determine plant species richness and community composition according to alleyway treatment. Early spring ensured unsown spring ephemeral species were recorded prior to the application of the cutting regimes. As all eight wildflower plots were managed the same in Year 1, four replicate wildflower plots were randomly selected and sampled, equivalent to the same number of control plots. From Year 2, the two different management strategies were randomly allocated, and all plots were surveyed. Six replicate 0.5 m × 0.5 m quadrats were randomly placed in each of the two wildflower strips within the survey area so that a total of 12 randomly placed quadrats were sampled from each plot at each sampling date.

All plant species present within quadrats were identified to species where possible, except for seven plants which were identified to the lowest rank possible (genus or family) and unsown Poaceae which were identified to family. Each species from within the quadrat was assigned a percentage cover score according to an 8‐point scale (1 = <1%, 2 = 1–5%, 3 = 6–10%, 4 = 11–20%, 5 = 21–40%, 6 = 41–60%, 7 = 61–80 and 8 = 81–100%). Bare soil and alleyway leaf litter (unattached) were recorded using absolute percentage cover values. The reproductive status of each species was also recorded as (i) vegetative only or had (ii) flower shoots present or budding, (iii) flowers open in bloom or (iv) seeds in formation, ripe or dehiscent. A 4‐point scale assigned the proportion of individuals at each stage (1 = 1%–25%, 2 = 26–50%, 3 = 51–75% and 4 = 76–100%) (Westbury et al., 2017).

To calculate the percentage cover scores for the resource classes (grasses and forbs), a reproductive percentage cover score was first estimated for each species surveyed within the quadrat. The reproductive percentage cover score was estimated from; (i) the reproductive scores per species and (ii) percentage cover score per species (as previously described). Firstly, reproductive scores for each species were back‐transformed to their mid‐point values to estimate the percentage of each of the four reproductive stages for each plant species (1 = 12%, 2 = 38%, 3 = 63% and 4 = 88%). Secondly, the estimated percentage cover each species occupied within the whole quadrat (0.5%, 2.5%, 8%, 15.5%, 30.5%, 50.5%, 70.5% or 90.5%) was multiplied by the estimated reproductive scores, to calculate the reproductive percentage cover scores for each species at each reproductive status. Once the reproductive percentage cover score for each species had been calculated, these scores were summed across all grasses or all forbs to give the percentage cover scores for the reproductive resource classes for each of the 10 groups (vegetative forbs, vegetative grasses, budding forbs, budding grasses, flowering forbs, flowering grasses, dehiscent forbs and dehiscent grasses). The non‐reproductive resource classes (bare soil and alleyway leaf litter) were scored using absolute percentage cover values.

### 2.4 Vegetation height and structural heterogeneity

The height of the alleyway vegetation was measured and the structural heterogeneity then determined by calculating the coefficient of variation for each alleyway sampling area. For this, a wooden disc of a standard diameter (30 cm) and weight (200 g) was dropped down a 1 m rule and the height it rested on the sward was recorded (Stewart et al., 2001). Twenty drop disc measurements were taken monthly from each alleyway from April to October during all study years. The mean vegetation height per plot was calculated and the coefficient of variation for each plot, expressed as a percentage, was calculated using the following formula:

$$\mathrm{CV}=\frac{\mu }{\sigma }\times 100$$

where *μ* is the standard deviation in drop disc measurements per plot and *σ* is the mean.

### 2.5 Statistical analysis

All statistical analyses were performed using RStudio Version 1.3.1056 (RStudio Team, 2015) for R version 4.0.2 (R Core Team, 2019). Data manipulation was carried out using the tidyr and dplyr packages (Wickham, 2020; Wickham et al., 2020). All colours for graphical representation were selected using RColorBrewer and assigning colorblindFriendly to true (Neuwirth, 2022).

#### 2.5.1 Species richness

Plant species richness was calculated for each quadrat. Unspecified taxa contain at least a single species, so were each counted as one species. A negative binomial generalised linear mixed effects model (GLMM) was fitted using lme4 (Bates et al., 2015) to infer differences in plant species richness between treatments, study years and their interaction. To account for the randomised block design, random intercepts were fitted for each orchard block (site). Model assumptions were visually checked by generating a QQ plot and checking for distribution about *x* = *y*. Stepwise reduction in the model was conducted and Wald chi‐squared tests using ANOVA function of car (Fox & Weisberg, 2019) assessed the significance of terms within the final model.

#### 2.5.2 Community composition

Differences in plant community composition between treatments, orchards, and study years were visualised using boral (Hui, 2016) and further investigated with mvabund (Wang et al., 2012). The establishment year was excluded from the analysis. First, an unconstrained negative binomial latent variable model was constructed in boral and the posterior medians plotted (Hui, 2016). The response variable was a multivariate‐vector matrix consisting of the cover scores (converted to the mid‐point as described above) for each plant species identified across all the plots during Years 2 and 3. Second, the same 72‐vector plant species abundance matrix was regressed against treatment, year and site, using a negative binomial multivariate GLM from the mvabund package (Wang et al., 2012). Null models were then fitted, and ANOVA performed with 999 bootstrapped resamples, with restricted permutations within orchard blocks (Simpson, 2019), to test for significance between models. Test statistics were generated via likelihood‐ratio test and the *p‐*value estimated via PIT‐trap resamples (Warton et al., 2017). Univariate GLMs were fitted for each plant species to investigate which displayed the strongest responses to alleyway treatment (Wang et al., 2012). The contribution of each plant species to the global treatment effect was determined by comparing the univariate test statistics to the total deviance due to treatment in the global model.

#### 2.5.3 Sown species

To assess the inherent differences in establishment success between wildflower strip management, cover values of the 14 sown species were converted from the eight‐point scale to a cover mid‐point score (1 = 1%, 2 = 3%, 3 = 8%, 4 = 15%, 5 = 30%, 6 = 50%, 7 = 70% and 8 = 90%) and regressed against treatment (SMWT and AMWT) and years (1, 2 and 3), including their interaction, using a negative binomial multivariate‐glm from the mvabund package (Wang et al., 2012). Once assumptions were visually verified, ANOVA was performed with restricted permutations. Test statistics were generated via likelihood‐ratio test and the *p*‐value estimated via Monte Carlo bootstrapped resamples, run 999 times. As above, species responses were explored using univariate GLMs (Wang et al., 2012).

#### 2.5.4 Vegetation height and structural heterogeneity

Differences in vegetation height and structural heterogeneity between treatments were determined by fitting linear mixed effects models (LMM) using lme4 (Bates et al., 2015). As before, the establishment year was modelled separately. A separate model was fitted for each response variable, vegetation height and coefficient of variation of vegetation height. All response variables were log transformed (*n* + 1) to account for their non‐normal distribution. The response was regressed against treatment for Year 1 and treatment × year for Years 2 and 3. Random intercepts were fitted for each orchard block (site) as well as for each sample date. Model assumptions were visually checked by generating a QQ plot and checking for distribution about *x* = *y*. Stepwise reduction in the model was conducted, and Wald chi‐squared tests using the ANOVA function of car (Fox & Weisberg, 2019) assessed the significance of terms within the final model. Pairwise comparisons between treatments were conducted using emmeans (Lenth et al., 2022).

#### 2.5.5 Resource provision

The differences in percentage cover of each resources class according to treatment were investigated using GLMM. The establishment year was modelled separately to the two subsequent years. The response was the percentage cover score assigned to each of the 10 cover classes (vegetative forbs, vegetative grasses, budding forbs, budding grasses, flowering forbs, flowering grasses, dehiscent forbs and dehiscent grasses). As the response was a continuous proportion, it was first logit transformed (Douma & Weedon, 2019; Warton & Hui, 2011) and then regressed against resource class, treatment and year, as well as their interactions. Orchard block was included as a random effect. The summary function verified the inclusion of each term in the model. To infer differences in resource class across the treatments, a null model was constructed in which the resource class × treatment interaction was removed. To determine whether treatment effects were consistent across years, a second null model was constructed in which the treatment × cover‐type × year, cover‐type × year and treatment × year interactions were removed. Null models were then compared with the maximal model using ANOVA. Pairwise comparisons between treatments were conducted using emmeans (Lenth et al., 2022).

## 3 RESULTS

A total of 72 plant species were identified across the 3‐year study. Irrespective of year, forbs species covered approximately one‐third of SMWT (37.6% cover) and AMWT (32.6%) alleyways, whereas only 16% of the control was occupied by forbs species. The control was instead dominated by bare ground (34.8%) with a patchy cover of grass species (28%). Grasses covered approximately a third of SMWT (31.9%) and AMWT (33.6%). In sown plots, irrespective of the year, the most dominant forb was the sown species *Plantago lanceolata*, representing 16.1% in SMWT and 17.0% in AMWT. *Crepis capillaris* (2.5%) and *Echium plantagineum* (1.8%) were the most abundant forbs in the unsown control, but no other species were consistently (>1%) recorded.

### 3.1 Species richness

**FIGURE 2:**
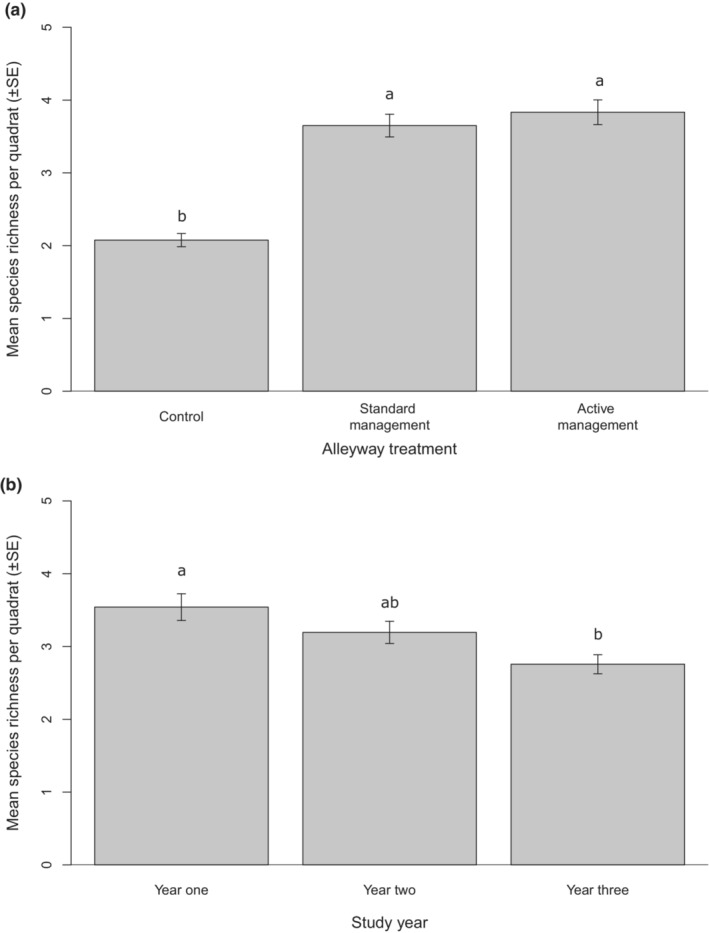
Mean species richness between (a) the three treatments; control, standard management wildflower treatment (SMWT) and active management wildflower treatment (AMWT), summed across Years and (b) study years, 1 (2017), 2 (2018) and 3 (2019), summed across treatments. Error bars represent ±1 SEM. Superscripts represent significant differences (Tukey's pairwise contrasts; *p* < .05).

The number of plant species within alleyways was influenced by treatment (GLMM: χ^2^ = 92.05, df = 2, *p* < .001) (Figure 2a) and study year (GLMM: χ^2^ = 22.76, df = 2, *p* < .001) (Figure 2b, Appendix B: Table B1.1). However, the treatment effect was consistent between years, as indicated by the non‐significant interaction (GLMM: χ^2^ = 9.18, df = 2, *p* = .06).

Irrespective of sampling year, alleyways established with wildflower strips under either cutting strategies contained almost double the number of plant species (3.65 ± 0.16 species per 0.25 m^2^ in SMWT and 3.83 ± 0.17 per 0.25 m^2^ in AMWT) compared with the unsown control alleyways (2.09 ± 0.10 species per 0.25 m^2^). There was no difference between the two wildflower treatments (Figure 2a and Appendix B: Tables B1.1 and B1.2). Irrespective of treatment, species richness steadily decreased during the 3‐year study, from a mean of 3.54 ± 0.18 species per 0.25 m^2^ in Year 1 to 2.76 ± 0.13 per 0.25 m^2^ in Year 3 (Figure 2b). There was no difference in plant species richness between Years 1 and 2 and Years 2 and 3.

### 3.2 Community composition

**FIGURE 3:**
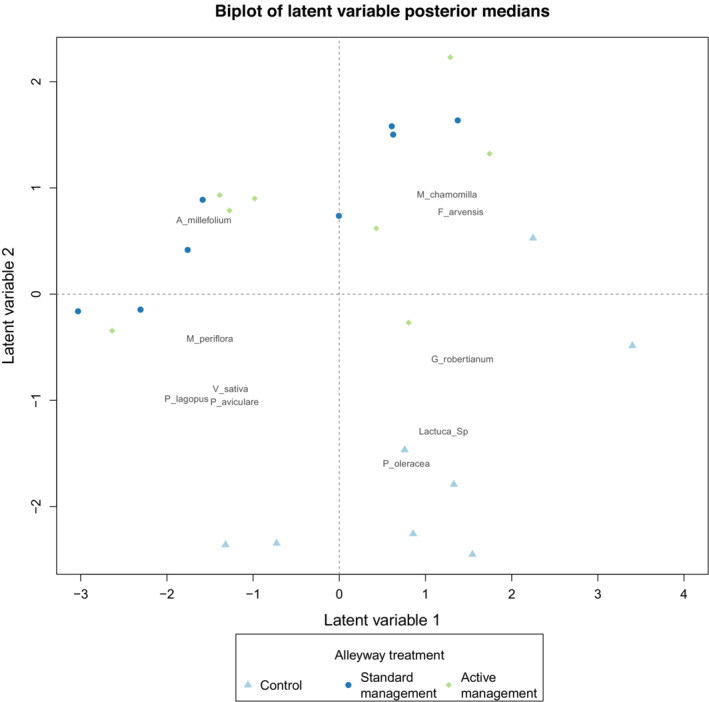
Biplot with 10 indicator species based on the negative binomial latent variable model (boral). The points correspond to site indices. Points are coloured to visualise community separation according to treatment, where control are light blue triangles, standard management wildflower treatment (SMWT) are dark blue circles, and active management wildflower treatment (AMWT) are green rhombi.

Visualisation of the plant communities in alleyways associated with the three different treatments indicates that the control treatment was markedly different from those managed with SMWT and AMWT (Figure 3 and Table 2).

**TABLE 2:** Species significantly different in abundance between treatments during Years 2 and 3 (2018 and 2019).

Species	Sown/unsown	Test statistic (LR)	Per cent of total treatment effect (%)	Mean % cover (±SE)		
				Control	SMWT	AMWT
*Plantago lanceolata*	Sown	44.3	7.7	–	16.8 (±5.1)	18.6 (±2.6)
*Dactylis glomerata*	Sown	38.4	6.4	–	7.0 (±1.5)	11.0 (±4.1)
*Achillea millefolium*	Sown	28.7	5.0	–	2.4 (±1.0)	1.2 (±0.5)
*Salvia verbenaca*	Sown	20.2	3.5	–	1.3 (±0.5)	0.2 (±0.1)
*Crepis capillaris*	Unsown	19.0	3.3	1.9 (±1.4)	0.9 (±0.8)	–

*Note*: The likelihood ratio for each univariate test is given with the percentage contribution of the total treatment affect. Mean percentage cover scores for each treatment, control, standard management wildflower treatment (SMWT) and active management wildflower treatment (AMWT), are specified.

Once established, the plant communities (based on percentage cover values per species for Years 2 and 3) varied between the three alleyway management treatments (anova.manyglm: LRT = 578.0, df = 2, *p* < .001) and this treatment effect remained consistent between Years 2 and 3 (anova.manyglm: LRT = 124.0, df = 2, *p* = .337). Five species contributed over a quarter of the total community treatment effect (26.1%) (Table 2). The sown forb *P. lanceolata* (accounting for 7.7% of the total treatment effect) was the dominant forb in both wildflower communities and was absent from the unsown control community. *Achillea millefolium* (5.0% of the total treatment effect) and *S. verbenaca* (3.5%) were also key components within both wildflower strip treatments but tended to be most abundant with SMWT. The sown grass species *D. glomerata* was present in both wildflower treatments (accounting for 6.6% of the total treatment effect) but was generally most abundant as part of the community in AMWT. The unsown forb *Crepis capillaris* was an important component of the control alleyway plant communities and was completely absent from the AMWT (accounting for 3.3% of the total treatment effect) (Figure 3 and Table 2).

### 3.3 Sown species

**FIGURE 4:**
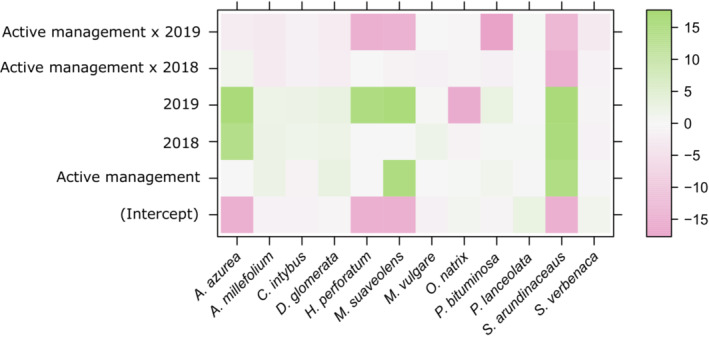
Percentage cover scores of sown species in response to management (standard management wildflower treatment: SMWT or active management wildflower treatment: AMWT) across the 3‐year study. The coefficients from mvabund model are plotted; no difference from the mean is white, an increase is green and a decrease is pink. The scale represents the mean change in the response from the predictor and therefore denotes the magnitude of the response, with the sign indicating direction.

Overall, the performance of the sown species based on percentage cover values did not vary between alleyway treatments (anova.manyglm: LRT = 19.69, df = 1, *p* = .082). However, the abundance of sown species varied between years (manyglm: LRT = 82.36, df = 2, *p* = .001). Moreover, the cutting treatments applied to the wildflower strips led to different responses of sown species abundance between treatments with time, as observed by the significant treatment × year effect (manyglm: LRT = 41.12, df = 2, *p* = .006). The consistent negative response of *Ononis natrix* across the length of the study was responsible for 49.5% of the total year effect within the model. Irrespective of treatment, the percentage cover of *O. natrix* decreased between years, from 3.0% cover (±0.5) in Year 1, to 0.9% (±0.3) in Year 2, to being absent from quadrat samples in the final year (Year 3). The different responses of *Psoralea bituminosa* to the alleyway treatments with time were responsible for 39.2% of the total treatment × year effect within the model. In the establishment year (Year 1), the percentage cover of *P. bituminosa* in the AMWT (1.5% ± 0.4) was three times that of SMWT (0.5% ± 0.3). In the AMWT, the percentage cover decreased across the 3 years until being absent from quadrat samples in the final year. Conversely, in the SMWT *P. bituminosa* cover increased to 0.7% (±0.4) in Year 2, to 5.8% (±2.7) by Year 3 (Figure 4 and Appendix B: Table B2).

### 3.4 Vegetation height and structural heterogeneity

**FIGURE 5:**
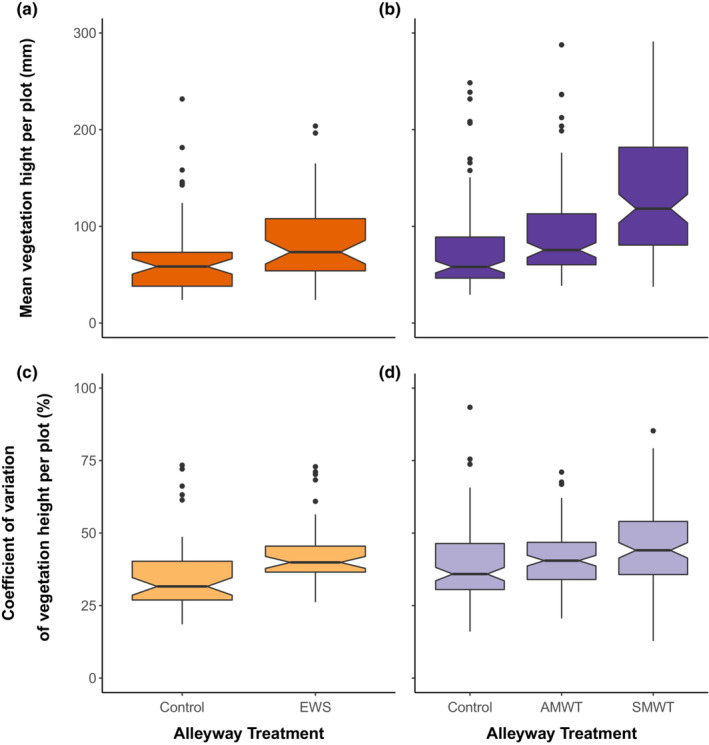
Mean height of vegetation in the alleyways between rows of *Citrus* trees in response to management: control and establishing wildflower strips EWS during Year 1, (a) and control and standard management wildflower treatment (SMWT) and active management wildflower treatment (AMWT) during Years 2 and 3, (b) Structural heterogeneity of the alleyway vegetation, calculated as the coefficient of variation of height, in response to management: control and EWS during Year 1, (c) and control and SMWT and AMWT during Years 2 and 3, (d) Where box represents the interquartile range and the notch indicates the 95% confidence interval about the median.

The height of the alleyway vegetation was increased by the wildflower strips during both the establishment year (ANOVA, χ^2^ = 7.78, df = 1, *p* = .005) and in subsequent years (ANOVA, χ^2^ = 124.08, df = 2, *p* < .001) (Figure 5). Vegetation height was 22% higher in plots where wildflower strips were establishing (84.1 mm ± 6.2) in comparison with the control plots (68.9 mm ± 6.4) (Year 1). During Years 2 and 3, vegetation height was 23% higher in AMWT plots (92.0 mm ± 4.5) and 84% higher in the SMWT (137.6 mm ± 6.2) in comparison with the control plots (74.5 mm ± 4.0). Furthermore, the wildflower strips enhanced structural heterogeneity both during the establishment year (ANOVA, χ^2^ = 15.36, df = 1, *p* < .001) and in subsequent years (ANOVA, χ^2^ = 124.08, df = 2, *p* < .001) (Figure 5). During the establishment year, structural heterogeneity was increased by 26.2% with the EWS (44.4% ± 2.5) compared with the control (35.1% ± 1.9). In Years 2 and 3, the SMWT (46.8% ± 1.6) enhanced structural heterogeneity by 17% relative to the control treatment (40.0% ± 1.4), whereas AMWT (41.0%) was similar to the control.

### 3.5 Resource provision

**FIGURE 6:**
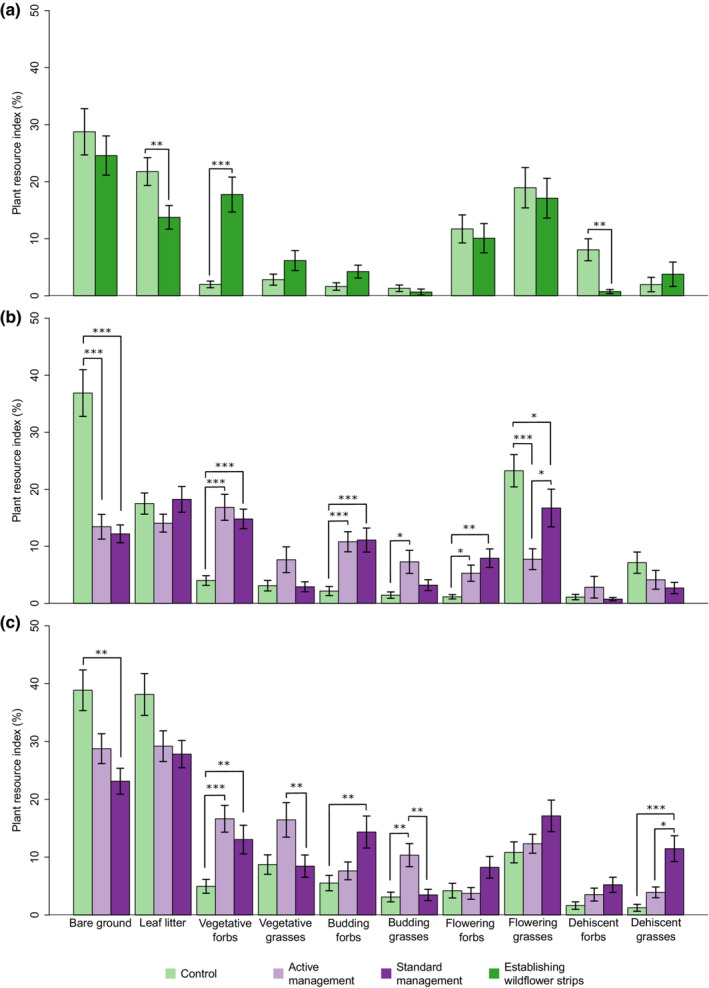
Plant resource index (%) by resource class, bare ground, leaf litter, vegetative forbs, vegetative grasses, budding forbs, budding grasses, flowering forbs, flowering grasses, dehiscent forbs and dehiscent grasses (irrespective of being sown/unsown) between treatments during (a) the establishment Year 1 (2017), (b) Year 2 (2018) and (c) Year 3 (2019). Error bars represent ±1 SEM. Superscripts denote significant differences in the plant resource index between treatments (Tukey's pairwise contrasts; *p* < .05).

During the establishment year (Year 1), the resources for natural enemies were influenced by alleyway treatment (ANOVA, χ^2^ = 64.77, df = 8, *p* < .001). This was driven by the lower cover of leaf litter and dehiscent forbs, coupled with a greater cover of vegetative forbs in the wildflower strips compared with control alleyways (Figure 6 and Appendix B: Table B3).

In Years 2 and 3, once the cutting treatments had been applied to the wildflower strips, resource provision was further influenced by treatment (ANOVA, χ^2^ = 329.79, df = 45, *p* < 0.001). Irrespective of the year, the cover of bare ground in the alleyways of the SMWT and AMWT was reduced by almost half compared with the control alleyways. Instead, SMWT and AMWT were associated with more than double the cover of vegetative and budding forbs than the control. AMWT was also associated with more than double the cover of vegetative and budding grasses compared with the control and SMWT, whereas the SMWT was associated with greater cover of flowering and dehiscent grasses. However, this treatment effect was not consistent for all resource classes across all study years (ANOVA, χ^2^ = 147.4, df = 29, *p* < .001) (Figure 6). The cover of bare ground and leaf litter increased in both the SMWT and AMWT between Years 2 and 3 along with increased cover of vegetative grasses. The SMWT was further associated with increased cover of forbs and grasses which had set seed in the SMWT in Year 3 (Figure 6 and Table 3).

**TABLE 3:** Effect of alleyway treatment, control, standard management wildflower treatment (SMWT) and active management wildflower treatment (AMWT), on plant provided resource for natural enemies during Years 2 and 3 (2018 and 2019).

Resource class	Study year	Percentage cover (%)			Control‐AMWT		Control‐SMWT		AMWT‐SMWT	
		Control	SMWT	AMWT	HSD	*p*‐value	HSD	*p*‐value	HSD	*p*‐value
Bare ground	2018	36.88 (±4.10)	12.19 (±1.57)	13.44 (±2.16)	6.22	**<.001**	6.27	**<.001**	0.05	.999
Bare ground	2019	38.85 (±3.51)	23.13 (±2.24)	28.75 (±2.58)	1.80	.169	3.18	**.004**	1.38	.351
Leaf litter	2018	17.50 (±1.85)	18.23 (±2.25)	14.06 (±1.57)	1.11	.507	3.18	**.004**	1.38	.351
Leaf litter	2019	38.13 (±3.61)	27.81 (±2.36)	29.19 (±2.65)	1.89	.142	1.88	.144	0.00	1.000
Vegetative forbs	2018	4.01 (±0.85)	14.80 (±1.73)	16.84 (±2.28)	−5.48	**<.001**	−5.20	**<.001**	0.27	.959
Vegetative forbs	2019	4.95 (±1.20)	13.04 (±2.48)	16.63 (±2.32)	−4.84	**<.001**	−3.29	**.003**	1.55	.266
Vegetative grasses	2018	3.11 (±0.92)	2.91 (±0.87)	7.66 (±2.26)	−1.45	.315	0.01	1.000	1.46	.312
Vegetative grasses	2019	8.72 (±1.69)	8.45 (±1.92)	16.45 (±2.99)	−2.16	.078	0.93	.622	3.09	**.006**
Budding forbs	2018	2.16 (±0.79)	11.10 (±2.10)	10.81 (±1.76)	−4.71	**<.001**	−4.35	**<.001**	0.36	.931
Budding forbs	2019	5.52 (±1.33)	14.34 (±2.77)	7.62 (±1.54)	−1.06	.542	−3.13	**.005**	−2.08	.095
Budding grasses	2018	1.45 (±0.56)	3.20 (±0.95)	7.28 (±2.02)	−2.58	**.027**	−1.00	.578	1.59	.251
Budding grasses	2019	3.11 (±0.84)	3.47 (±0.96)	10.34 (±1.99)	−3.28	**.003**	0.02	1.000	3.29	**.003**
Flowering forbs	2018	1.16 (±0.38)	7.92 (±1.62)	5.30 (±1.42)	−2.36	**.048**	−3.51	**.001**	−1.15	.482
Flowering forbs	2019	4.21 (±1.27)	8.25 (±1.87)	3.73 (±1.00)	0.08	.996	−1.73	.196	−1.81	.167
Flowering grasses	2018	23.25 (±2.83)	16.72 (±3.31)	7.75 (±1.82)	5.61	**<.001**	2.79	.015	−2.82	**.013**
Flowering grasses	2019	10.83 (±1.81)	17.14 (±2.73)	12.32 (±1.63)	−0.95	.607	−1.88	.146	−0.92	.626
Seeding forbs	2018	1.11 (±0.47)	0.75 (±0.29)	2.84 (±1.90)	−0.46	.889	0.08	.997	0.54	.851
Seeding forbs	2019	1.62 (±0.65)	5.22 (±1.31)	3.53 (±1.12)	−1.11	.506	−2.01	.110	−0.90	.642
Seeding grasses	2018	7.14 (±1.86)	2.69 (±0.98)	4.13 (±1.65)	1.50	.290	1.96	.123	0.46	.891
Seeding grasses	2019	1.25 (±0.60)	11.47 (±2.24)	3.91 (±0.94)	−1.95	.125	−4.85	**<.001**	−2.90	**.010**

*Note*: The mean cover of each resource class (±SE) is presented along with Tukey's pairwise comparison between alleyway treatments (Control‐AMWT, Control‐SMWT and AMWT‐SMWT). Significant differences are given in bold.

## 4 DISCUSSION

This is the first time that the performance of native perennial forbs and tussock‐forming grasses has been assessed in Mediterranean orchard systems. One sown grass and eight of the 12 forbs performed consistently throughout the study and hence are recommended for inclusion in the further use of Mediterranean seed mixes. The wildflower strips supported distinct plant communities compared with the unsown control alleyways and were associated with greater plant species richness. Importantly, the management treatments (standard or active) applied to the wildflower strips differentially influenced the cover of sown species, resources provided and the height and structure of the alleyway vegetation.

The active management of wildflower strips (AMWT) favoured plant species which could reproduce vegetatively, including *Mentha suaveolens*, and two sown grass species, *D. glomerata* and *S. arundinaceus*. In situations where water availability can limit seed germination and successful establishment of perennial species, the ability to reproduce vegetatively can be advantageous (Clary, 2008). When under stressed conditions, such as water stress, species which can reproduce via both means show preference for vegetative reproduction; as a short‐term strategy, it is considered a key driver to dominance in perennial communities (Yang & Kim, 2016). However, although *A. millefolium* can reproduce vegetatively via rhizomes (Kannangara & Field, 1985), it did not increase during the study.

The promotion of species capable of vegetative growth due to regular cutting can competitively exclude species unable to reproduce via this strategy (Bricca et al., 2020). For example, despite the initial successful establishment of *P. bituminosa* and *S. verbenaca* in all sown orchard alleyways, cover values with AMWT were lower compared with the standard management of annual cutting (SMWT). Competitive exclusion through increased grass abundance and the associated lower values of bare ground in SMWT is an ongoing issue for newly created wildflower strips (Westbury et al., 2008). However, the higher species richness associated with SMWT is expected to offer more stability, enhancing community resilience to environmental change (Tilman et al., 2006). In contrast, the unsown control alleyways exhibited greater percentage cover values of bare ground and leaf litter than alleyways established with wildflower strips. The regular cutting associated with this conventional approach is expected to reduce the ability of many plant species to flower and set seed, including spring annuals, resulting in greater values of bare ground. Cutting Mediterranean plant communities typically increases the abundance of annual grasses and forbs, which are able to exploit the newly formed germination niches, and also favours prostrate species such as knotgrass (*Polygonum aviculare*) (Merou et al., 2013). However, when disturbance is frequent, this leads to the loss of individuals and increases the amount of bare ground (Souther et al., 2019).

Wildflower strips were associated with an increased height of alleyway vegetation relative to the control, but only the SMWT increased the structural heterogeneity of the alleyway vegetation. Importantly, greater structural heterogeneity can reduce competition between natural enemies (Chesson, 2000). For example, in simple homogeneous habitat phytophage diversity and abundance tends to be limited and hence competition for shared resource between predators increases (Woodcock et al., 2009). Predatory beetle richness is strongly positively related to vegetation structural complexity (Woodcock et al., 2007). High structural heterogeneity within wildflower strips is typically associated with access to resource (Westbury et al., 2017), including parasitoids (Mockford et al., 2022) and farmland birds (Vickery et al., 2001).

Irrespective of management treatment, the wildflower strips enhanced the abundance of plant resources and increased vegetation height. Both SMWT and AMWT were associated with reduced covers of bare ground and leaf litter, whilst the cover of forbs and grasses in the vegetative and budding stages were increased. In contrast, alleyways managed with the conventional farm management in the control were associated with a greater cover of forbs and grasses that had already set seed, such as *Scorpius muricatus* (Fabaceae), *Astragalus sesameus* (Fabaceae) and *Erodium cicutarium* (Geraniaceae) suggesting much of the flowering occurred in early spring, before sampling in May. Many natural enemies require plant resources, such as nectar and pollen, to complete their lifecycles (Wäckers et al., 2005). Forbs in the cropped environment support important groups of natural enemies, such as syrphids and coccinellids (González et al., 2022). As designed, the combined flowering period of the wildflower strips was extended in SMWT and AMWT compared with the control.

The vegetative stage of plants supports alternative prey/host and honeydew‐producing species (Gómez‐Marco et al., 2016) and provides shelter and refuge for natural enemies (Aucejo et al., 2003; Thomas et al., 1991; Woodcock et al., 2005). The effects of which are strongly influenced by vegetation height and structure (Atkinson et al., 2005; Woodcock et al., 2007). Indeed, the structural heterogeneity of vegetation is a strong driver of invertebrate assemblages and is enhanced through plant trait and functional group diversity (Woodcock et al., 2009). However, the wildflower strips, which were designed to include a diversity of plant growth forms, phenology and morphology, only enhanced the structural heterogeneity when allowed to grow throughout the season, as in SMWT. By reducing competition between higher trophic levels through diversification of prey and host items (Chesson, 2000), structural heterogeneity can enhance predatory species richness (Woodcock et al., 2009). In the final year of the study, SMWT was associated with an increased cover of flowering and dehiscent grasses, likely due to unsown grass species exploiting available niches (Storkey & Westbury, 2007). However, grass pollen is an important food source for Neuroptera (Alcalá Herrera et al., 2020), many of which are implicit in pest regulation, such as *Chrysoperla carnea* (Stephens) (Neuroptera: Chrysopidae) and *Conwentzia psociformis* (Curtis) (Neuroptera: Coniopterygidae) (Garcia Marí & Bru, 2008).

Four sown species did not perform well during the 3‐year study, irrespective of management treatment: *Hypericum perforatum*, *O. natrix*, *T. vulgare* and *H. stoechas*. Both *H. perforatum* and *T. vulgare* were only recorded in one plot, whilst *H. stoechas* was not recorded in any quadrat samples, nor observed whilst conducting surveys. *Helichrysum stoechas* also failed to establish in suitability trials for erosion control in the Mediterranean (Oliveira et al., 2012). Despite the initial establishment success of *O. natrix*, it was not recorded from any sown wildflower alleyways by Year 3. Atallah et al. (2008) observed *O. natrix* only as scattered individuals in open Mediterranean shrubland vegetation. This suggests that *O. natrix* may have been outcompeted. The establishment of *H. perforatum*, *O. natrix*, *T. vulgare* and *H. stoechas* might have also been influenced by the sowing rates used (Stevenson et al., 1995). Higher sowing rates can increase success by reducing latent factors confounding establishment, ensuring at least a few individuals can persist (Jaksetic et al., 2018; Stevenson et al., 1995). Further investigation is therefore required to determine if increasing the initial sowing rate might enhance the successful establishment of poor‐performing species.

Several unsown species recorded in the alleyways are known to support non‐pest alternative prey and/or hosts as well as their predators and parasitoids (Bertolaccini et al., 2011; Gómez‐Marco et al., 2016). *Medicago truncatul*a and *Trifolium glomeratum* were found across all treatments and *Trifolium scabrum* was recorded only in association with SMWT. However, the alleyways also supported plant species which may harbour potential pest species. *Malva sylvestris* and *Malva parviflora* were associated with SMWT and AMWT, which were likely stimulated by the disturbance of the seedbed in preparation for sowing (Schutte et al., 2014). AMWT was also associated with the unsown species *Solanum nigrum*, which is considered a problem species in *Citrus* (Celepci et al., 2017; Ferreira & Sousa, 2011). It is associated with the *Citrus* pest species *Planococcus citri* (Risso) (Hemiptera: Pseudococcidae), *Tetranychus urticae* Koch and *Tetranychus evansi* Baker (Acari: Tetranychidae) (Aucejo et al., 2003; Celepci et al., 2017; Ferreira & Sousa, 2011). *Solanum nigrum* is an annual species which germinates in late spring to early summer and performs best in unshaded open locations (Keeley & Thullen, 1989; Roberts & Lockett, 1978). Disturbance of the seedbed (Schutte et al., 2014), coupled with the regular cutting in AMWT (Merou et al., 2013; Souther et al., 2019), enabled this fast growing, prostrate annual to establish (Keeley & Thullen, 1989). In contrast, despite cultivation, vegetation in SMWT was associated with lower values of bare ground and therefore a denser sward leading to lower cover values of *S. nigrum*. Overall, despite *S. nigrum* in AMWT, it was the control treatment that was associated with the greatest range of plant species considered problematic (Celepci et al., 2017; Ferreira & Sousa, 2011).

Many unsown species can provide additional benefits, especially carbohydrates which can enhance biological control services in orchards (Tena et al., 2015). For example, despite its pest status in arable systems, *P. aviculare* provides nectar‐producing flowers in otherwise carbohydrate‐limited orchards (Mockford et al., 2022). The presence of unsown species in the soil seedbank could therefore be an important supplementary resource. Nonetheless, such species might be highly site‐specific, or may not provide consistent year‐on‐year resources (Fiedler & Landis, 2007). In contrast, sown perennial wildflower strips, left uncut through the season as in SMWT, increase the availability of plant resources expected to support natural enemies and by design extend the period for which resources are available. Further research is now required to assess the effect on natural enemies and the delivery of pest regulation services to the crop.

## 5 CONCLUSIONS

The study demonstrates for the first time that a specifically designed perennial seed mix can enhance plant resources expected to support natural enemies in *Citrus*. However, to maximise the benefits, we recommend managing with cutting once per year in May. From this study, eight sown forbs species, *A. azurea*, *S. verbenaca*, *P. bituminosa*, *M. suaveolens*, *P. lanceolate*, *M. vulgare*, *A. millefolium*, *C. intybus* and one tussock‐forming grass species, *D. glomerata* have been identified for further use in Mediterranean wildflower strips. Two unsown legumes, *M. truncatula* and *T. glomeratum*, performed consistently across all treatments and should be investigated further for their suitability. In turn, the study provides tools which will facilitate the development and adoption of perennial wildflower strips in other Mediterranean orchards systems, for example almonds, persimmons and pomegranates, where there is a real need to support sustainable production.

## AUTHOR CONTRIBUTIONS

**Alice Mockford:** Data curation (equal); formal analysis (equal); investigation (equal); methodology (equal); validation (equal); visualization (equal); writing – original draft (equal). **Alberto Urbaneja:** Methodology (equal); supervision (equal); validation (equal); writing – review and editing (equal). **Kate Ashbrook:** Methodology (equal); supervision (equal); validation (equal); writing – review and editing (equal). **Duncan B. Westbury:** Conceptualization (equal); funding acquisition (equal); methodology (equal); supervision (equal); validation (equal); writing – review and editing (equal).

## FUNDING INFORMATION

The project leading to this research was funded by the University of Worcester, Waitrose & Partners, and Primafruit Ltd.

### OPEN RESEARCH BADGES

This article has earned an Open Data badge for making publicly available the digitally‐shareable data necessary to reproduce the reported results. The data is available at https://doi.org/10.5061/dryad.cjsxksnbh.

## Data Availability

The data that support the findings of this study are openly available in Dryad at https://doi.org/10.5061/dryad.cjsxksnbh.

## APPENDIX A

### A.1 Detailed site description

The study was conducted in four sweet orange (*Citrus sinensis* Osbeck cv. Navel) orchards within three farms (sites), Madre del Agua, La Calvilla and Montepinos, at two different localities in the province of Huelva, south‐west Andalusia, Spain. Huelva is an important *Citrus*‐growing region of Spain, characterised by a sub‐tropical Mediterranean climate, with an average annual temperature of 17°C and annual precipitation of 525 mm (AEMET, 2020). The farm of Montepinos was situated in the north of Huelva (37°47′43.21 N 6°56′21.11 W) at 351 m elevation and characterised by clay soil. The farms Madre del Agua (37°26′27.80″N 7°9′55.73″W) and La Calvilla (37°24′10.95″N 7°3′42.67″W) were situated in the south of Huelva at 159 m (Madre del Agua) and 153 m (La Calvilla) of elevation and both characterised by sandy soil. The two localities were 42.59 km apart.

Conventional management practices of the orchard alleyways included regular cutting of the naturally occurring vegetation to <5 cm in height, four to five times annually. During the winter months, the naturally occurring vegetation of the alleyways was allowed to grow until the first cut of the year. Debris pruned from the *Citrus* trees, typically in late spring, was discarded in the alleyways and a tractor‐mounted disc mulcher used to shred it. Shredded plant material was then left in situ.

## APPENDIX B

### B.1 The influence of alleyway treatment on plant species richness within the alleyways

**TABLE B1.1:** Pairwise comparison of plant species richness recorded from the alleyways between rows of *Citrus* trees applied with the three different treatments in the alleyways: control, standard management wildflower treatment (standard) and active management wildflower treatment (active) and between the 3 years of the study: 1 (2017), 2 (2018) and 3 (2019).

Pair	Test	Test statistic	*p*‐value
Control‐standard	Tukey	−7.442	<.001
Control‐active	Tukey	−9.053	<.001
Standard‐active	Tukey	−1.589	.25
2017–2018	Tukey	2.779	.015
2017–2019	Tukey	4.420	<.001
2018–2019	Tukey	1.779	.177

**TABLE B1.2:** Mean plant richness per quadrat (±SE) of all genera, sown genera and unsown genera recorded from the three different treatments, control, standard management wildflower treatment and active management wildflower treatment, across each of the three study years, 2017, 2018 and 2019.

Alleyway treatment	2017	2018	2019
Control	2.40 (±0.15)	1.98 (±0.17)	1.90 (±0.17)
Standard management wildflower	5.25 (±0.23)	4.23 (±0.28)	3.21 (±0.24)
Active management wildflower	3.96 (±0.25)	4.02 (±0.29)	3.19 (±0.22)

### B.2 The influence of wildflower strip management on sown species abundance

**TABLE B2:** Sown species responses to the two wildflower strip management treatments, standard management (SMWT) and active management (AMWT), over the 3‐year study period, 2017, 2018 and 2019.

Species	Treatment × year effect		Study year	Percentage cover (%)	
	LRT	Variation within model (%)		AMWT	SMWT
*P. bituminosa*	16.13	39.23	2017	1.46 (±0.35)	0.46 (±0.25)
			2018	0.56 (±0.29)	0.73 (±0.36)
			2019	–	5.75 (±2.71)
*M. suaveolens*	6.49	15.78	2017	0.88 (±0.27)	–
			2018	0.33 (±0.23)	–
			2019	2.19 (±1.09)	1.67 (±0.75)
*S. arundinaceaus*	6.07	14.75	2017	0.58 (±0.26)	–
			2018	0.88 (±0.42)	1.58 (±0.72)
			2019	6.77 (±1.80)	2.15 (±1.24)
*D. glomerata*	4.73	11.51	2017	9.25 (±2.26)	0.63 (±0.44)
			2018	9.98 (±2.99)	4.65 (±1.40)
			2019	12.04 (±2.74)	9.35 (±2.31)
*A. millefolium*	2.96	7.20	2017	2.67 (±0.91)	0.33 (±0.13)
			2018	1.48 (±0.89)	2.69 (±1.23)
			2019	0.96 (±0.66)	2.06 (±0.83)
*S. verbenaca*	2.51	6.11	2017	2.17 (±0.56)	3.17 (±2.06)
			2018	0.25 (±0.12)	1.10 (±0.55)
			2019	0.06 (±0.06)	1.48 (±0.62)
*A. azurea*	0.79	1.92	2017	–	–
			2018	1.10 (±1.04)	0.31 (±0.31)
			2019	0.31 (±0.31)	2.50 (±1.77)
*P. lanceolata*	0.44	1.04	2017	10.54 (±2.11)	12.96 (±2.87)
			2018	20.83 (±2.73)	20.35 (±2.48)
			2019	16.31 (±2.85)	13.31 (±3.54)
*M. vulgare*	0.40	0.97	2017	0.25 (±0.12)	0.25 (±0.12)
			2018	0.33 (±0.23)	1.38 (±1.06)
			2019	0.31 (±0.31)	0.33 (±0.23)
*C. intybus*	0.33	0.80	2017	0.13 (±0.09)	0.33 (±0.24)
			2018	0.17 (±0.17)	1.63 (±0.80)
			2019	0.31 (±0.31)	3.06 (±1.54)
*O. natrix*	0.28	0.69	2017	3.71 (±0.76)	2.46 (±0.58)
			2018	0.79 (±0.33)	0.98 (±0.44)
			2019	–	–
*H. perforatum*	0.00	0.01	2017	–	–
			2018	–	–
			2019	–	0.94 (±0.69)

*Note*: The mean cover of each sown species (±SE) is presented, as well as the percentage contribution to the total treatment × year effect of the manyglm model, as calculated from the LRT test statistic.

### B.3 The influence of alleyway management on sown plant provided resource for natural enemies

**TABLE B3:** Effect of alleyway treatment, control and establishing wildflower strips (EWS), on plant provided resource for natural enemies during Year 1 (2017).

Resource class	Study year	Percentage cover (%)		Tukey's	
		Control	EWS	HSD	*p‐*value
Bare soil	2017	28.75 (±4.06)	24.58 (±2.43)	0.98	.327
Leaf litter	2017	21.77 (±2.43)	13.75 (±1.46)	2.62	.009
Vegetative forbs	2017	2.00 (±0.58)	17.75 (±2.16)	−6.41	<.001
Vegetative grasses	2017	2.82 (±0.97)	6.17 (±1.24)	−1.69	.091
Budding forbs	2017	1.62 (±0.66)	4.24 (±0.79)	−1.65	.099
Budding grasses	2017	1.32 (±0.57)	0.65 (±0.37)	0.53	.596
Flowering forbs	2017	11.72 (±2.46)	10.09 (±1.81)	0.51	.613
Flowering grasses	2017	18.95 (±3.54)	17.11 (±2.46)	0.61	.544
Seeding forbs	2017	8.06 (±1.93)	0.74 (±0.26)	3.28	.001
Seeding grasses	2017	1.96 (±1.26)	3.78 (±1.51)	−0.47	.638

*Note*: The mean cover of each resource class (±SE) is presented along with the Tukey's pairwise comparison between alleyway treatments (control‐EWS).

## References

[ece310285-bib-0001] AEMET . (2020). Standard climate values. https://www.aemet.es/En/Serviciosclimaticos/Datosclimatologicos/Valoresclimatologicos

[ece310285-bib-0002] Aguilar‐Fenollosa, E. , Ibáñez‐Gual, M. V. , Pascual‐Ruiz, S. , Hurtado, M. , & Jacas, J. A. (2011). Effect of ground‐cover management on spider mites and their phytoseiid natural enemies in clementine mandarin orchards (II): Top‐down regulation mechanisms. Biological Control, 59(2), 171–179. 10.1016/j.biocontrol.2011.06.013

[ece310285-bib-0003] Albrecht, M. , Kleijn, D. , Williams, N. M. , Tschumi, M. , Blaauw, B. R. , Bommarco, R. , Campbell, A. J. , Dainese, M. , Drummond, F. A. , Entling, M. H. , Ganser, D. , Arjen de Groot, G. , Goulson, D. , Grab, H. , Hamilton, H. , Herzog, F. , Isaacs, R. , Jacot, K. , Jeanneret, P. , … Sutter, L. (2020). The effectiveness of flower strips and hedgerows on pest control, pollination services and crop yield: A quantitative synthesis. Ecology Letters, 23(10), 1488–1498. 10.1111/ele.13576 32808477 PMC7540530

[ece310285-bib-0004] Alcalá Herrera, R. , Fernández Sierra, M. L. , & Ruano, F. (2020). The suitability of native flowers as pollen sources for *Chrysoperla lucasina* (Neuroptera: Chrysopidae). PLoS One, 15(10), e0239847. 10.1371/journal.pone.0239847 33095792 PMC7584243

[ece310285-bib-0005] Atallah, T. , Rizk, H. , Cherfane, A. , Daher, F. B. , el‐Alia, R. , de Lajuide, P. , & Hajj, S. (2008). Distribution and nodulation of spontaneous legume species in grasslands and shrublands in Mediterranean Lebanon. Arid Land Research and Management, 22(2), 109–122. 10.1080/15324980801957978

[ece310285-bib-0006] Atkinson, P. W. , Fuller, R. J. , Vickery, J. A. , Conway, G. J. , Tallowin, J. R. B. , Smith, R. E. N. , Haysom, K. A. , Ings, T. C. , Asteraki, E. J. , & Brown, V. K. (2005). Influence of agricultural management, sward structure and food resources on grassland field use by birds in lowland England. Journal of Applied Ecology, 42(5), 932–942. 10.1111/j.1365-2664.2005.01070.x

[ece310285-bib-0007] Aucejo, S. , Foo, M. , Gimeno, E. , Gomez Cadenas, A. , Monfort, R. , Obiol, F. , Prades, E. , Ramis, M. , Ripolles, J. L. , Tirado, V. , Zaragoza, L. , Jacas, J. A. , & Martinez Ferrer, M. T. (2003). Management of *Tetranychus urticae* in citrus in Spain: Acarofauna associated to weeds, *integrated control in citrus fruit crops* . IOBC WPRS Bulletin, 26(6), 213–220.

[ece310285-bib-0008] Bates, D. , Mächler, M. , Bolker, B. , & Walker, S. (2015). Fitting linear mixed‐effects models using {lme4}. Journal of Statistical Software, 67(1), 1–48. 10.18637/jss.v067.i01

[ece310285-bib-0009] Berk, Z. (2016). Citrus fruit processing. Academic Press.

[ece310285-bib-0010] Bertolaccini, I. , Núñez‐Pérez, E. , & Tizado, E. J. (2011). Alternative plants hosts of legume aphids and predators in the province of León, Spain. Ciencia e investigación Agraria, 38(2), 233–242. 10.4067/s0718-16202011000200009

[ece310285-bib-0011] Bianchi, F. J. J. A. , Schellhorn, N. A. , & Cunningham, S. A. (2013). Habitat functionality for the ecosystem service of pest control: Reproduction and feeding sites of pests and natural enemies. Agricultural and Forest Entomology, 15(1), 12–23. 10.1111/j.1461-9563.2012.00586.x

[ece310285-bib-0012] Bricca, A. , Tardella, F. M. , Tolu, F. , Goia, I. , Ferrara, A. , & Catorci, A. (2020). Disentangling the effects of disturbance from those of dominant tall grass features in driving the functional variation of restored grassland in a sub‐Mediterranean context. Diversity, 12(1), 11.

[ece310285-bib-0013] Bugg, R. L. , & Waddington, C. (1994). Using cover crops to manage arthropod pests of orchards: A review. Agriculture, Ecosystems and Environment, 50(1), 11–28. 10.1016/0167-8809(94)90121-X

[ece310285-bib-0014] Campbell, A. J. , Biesmeijer, J. C. , Varma, V. , & Wäckers, F. L. (2012). Realising multiple ecosystem services based on the response of three beneficial insect groups to floral traits and trait diversity. Basic and Applied Ecology, 13(4), 363–370. 10.1016/j.baae.2012.04.003

[ece310285-bib-0015] Campbell, J. A. , Wilby, A. , Sutton, P. , & Wäckers, F. (2017). Getting more power from your flowers: Multi‐functional flower strips enhance pollinators and Pest control agents in apple orchards. Insects, 8(3), 101. 10.3390/insects8030101 28930157 PMC5620721

[ece310285-bib-0016] Cardinale, B. J. , Duffy, J. E. , Gonzalez, A. , Hooper, D. U. , Perrings, C. , Venail, P. , Narwani, A. , Mace, G. M. , Tilman, D. , Wardle, D. A. , Kinzig, A. P. , Daily, G. C. , Loreau, M. , Grace, J. B. , Larigauderie, A. , Srivastava, D. S. , & Naeem, S. (2012). Biodiversity loss and its impact on humanity. Nature, 486, 59–67. 10.1038/nature11148 22678280

[ece310285-bib-0017] Carvell, C. , Westrich, P. , Meek, W. R. , Pywell, R. F. , & Nowakowski, M. (2006). Assessing the value of annual and perennial forage mixtures for bumblebees by direct observation and pollen analysis. Apidologie, 37, 326–240. 10.1051/apido

[ece310285-bib-0018] Cassman, K. G. , & Grassini, P. (2020). A global perspective on sustainable intensification research. Nature Sustainability, 3(4), 262–268. 10.1038/s41893-020-0507-8

[ece310285-bib-0019] Celepci, E. , Uygur, S. , Kaydan, M. B. , & Uygur, N. (2017). Mealybug (Hemiptera: Pseudococcidae) species on weeds in *citrus* (Rutaceae) plantations in Çukurova plain, Turkey. Turkish Bulletin of Entomology, 7, 15–21. 10.16969/teb.14076

[ece310285-bib-0020] Chesson, P. (2000). Mechanisms of maintenance of species diversity. Annual Review of Ecology and Systematics, 31(1), 343–366.

[ece310285-bib-0021] Clary, J. (2008). Rainfall seasonality determines annual/perennial grass balance in vegetation of Mediterranean Iberian. Plant Ecology, 195, 13–20.

[ece310285-bib-0022] Douma, J. C. , & Weedon, J. T. (2019). Analysing continuous proportions in ecology and evolution: A practical introduction to beta and Dirichlet regression. Methods in Ecology and Evolution, 10(9), 1412–1430. 10.1111/2041-210X.13234

[ece310285-bib-0023] Duru, M. , Therond, O. , Martin, G. , Martin‐Clouaire, R. , Magne, M. A. , Justes, E. , Journet, E. P. , Aubertot, J. N. , Savary, S. , Bergez, J. E. , & Sarthou, J. P. (2015). How to implement biodiversity‐based agriculture to enhance ecosystem services: A review. Agronomy for Sustainable Development, 35(4), 1259–1281. 10.1007/s13593-015-0306-1

[ece310285-bib-0024] Englund, G. , & Cooper, S. D. (2003). Scale effects and extrapolation in ecological experiments. Advances in Ecological Research, 33, 161–213. 10.1016/s0065-2504(03)33011-9

[ece310285-bib-0025] FAO . (2021). Citrus fruit statistical compendium 2020. FAO.

[ece310285-bib-0026] Ferreira, M. A. , & Sousa, S. E. (2011). Hosts and distribution of the spider mite *Tetranychus evansi* (Acari: Tetranychidae) in Portugal. Acta Horticulturae, 917, 133–136. 10.17660/ActaHortic.2011.917.16

[ece310285-bib-0027] Fiedler, A. K. , & Landis, D. A. (2007). Attractiveness of Michigan native plants to arthropod natural enemies and herbivores. Environmental Entomology, 36(4), 751–765. 10.1093/ee/36.4.751 17716466

[ece310285-bib-0028] Fox, J. , & Weisberg, S. (2019). An {R} companion to applied regression (third). Sage. https://socialsciences.mcmaster.ca/jfox/Books/Companion/

[ece310285-bib-0029] Frank, S. D. , Shrewsbury, P. M. , & Esiekpe, O. (2008). Spatial and temporal variation in natural enemy assemblages on Maryland native plant species. Environmental Entomology, 37(2), 478–486. 18419920 10.1603/0046-225x(2008)37[478:satvin]2.0.co;2

[ece310285-bib-0030] Garcia Marí, F. , & Bru, P. (2008). Seasonal and spatial population trend of predatory insects in eastern‐Spain citrus orchards. https://www.researchgate.net/publication/263964840

[ece310285-bib-0031] Girling, R. D. , Breeze, T. D. , & Garrat, M. P. (2022). Advancing conservation biological control as a component of IPM of horticultural crops. In R. Collier (Ed.), Improving integrated pest management in horticulture. Burleigh Dodds.

[ece310285-bib-0032] Gómez‐Marco, F. , Urbaneja, A. , & Tena, A. (2016). A sown grass cover enriched with wild forb plants improves the biological control of aphids in citrus. Basic and Applied Ecology, 17(3), 210–219. 10.1016/j.baae.2015.10.006

[ece310285-bib-0033] González, E. , Bianchi, F. J. J. A. , Eckerter, P. W. , Pfaff, V. , Weiler, S. , & Entling, M. H. (2022). Ecological requirements drive the variable responses of wheat pests and natural enemies to the landscape context. Journal of Applied Ecology, 59(2), 444–456. 10.1111/1365-2664.14062

[ece310285-bib-0034] Gurr, G. M. , Wratten, S. D. , Landis, D. A. , & You, M. (2017). Habitat management to suppress Pest populations: Progress and prospects. Annual Review of Entomology, 62(1), 91–109. 10.1146/annurev-ento-031616-035050 27813664

[ece310285-bib-0035] Haaland, C. , Naisbit, R. E. , & Bersier, L. F. (2011). Sown wildflower strips for insect conservation: A review. Insect Conservation and Diversity, 4(1), 60–80. 10.1111/j.1752-4598.2010.00098.x

[ece310285-bib-0036] Hui, F. K. C. (2016). Boral – Bayesian ordination and regression analysis of multivariate abundance data in R. Methods in Ecology and Evolution, 7(6), 744–750. 10.1111/2041-210X.12514

[ece310285-bib-0037] Isaacs, R. , Tuell, J. , Fiedler, A. , Gardiner, M. , & Landis, D. (2009). Maximizing arthropod‐mediated ecosystem services in agricultural landscapes: The role of native plants. Frontiers in Ecology and the Environment, 7(4), 196–203. 10.1890/080035

[ece310285-bib-0038] Jacas, J. A. , & Aguilar‐Fenollosa, E. (2013). Effect of ground cover management on Thysanoptera (thrips) in clementine mandarin orchards. Journal of Pest Science, 86, 469–481. 10.1007/s10340-013-0494-x

[ece310285-bib-0039] Jacas, J. A. , & Urbaneja, A. (2010). Biological control in citrus in Spain: From classical to conservation biological control. In C. Aurelio & K. G. Mukerji (Eds.), Integrated management of arthropod pests and insect borne diseases (pp. 61–72). Springer. 10.1007/978-90-481-8606-8

[ece310285-bib-0040] Jaksetic, N. , Foster, B. L. , Bever, J. D. , Schwarting, J. , & Alexander, H. M. (2018). Sowing density effects and patterns of colonization in a prairie restoration. Restoration Ecology, 26(2), 245–254. 10.1111/rec.12550

[ece310285-bib-0041] Kannangara, H. W. , & Field, R. J. (1985). Growth of seedling *Achillea millefolium* L. (yarrow) in association with pea (*Pisum sativum* L.). Weed Research, 25(5), 355–361. 10.1111/j.1365-3180.1985.tb00656.x

[ece310285-bib-0042] Keeley, P. E. , & Thullen, R. J. (1989). Growth and competition of black nightshade (*Solanum nigrum*) and palmer Amaranth (*Amaranthus palmeri*) with cotton (*Gossypium hirsutum*). Weed Science, 37(3), 326–334.

[ece310285-bib-0043] Lenth, R. V. , Bolker, B. , Buerkner, P. , Giné‐Vázquez, I. , Herve, M. , Jung, M. , Love, J. , Miguez, F. , Riebl, H. , & Singmann, H. (2022). emmeans: Estimated marginal means, aka least‐squares means. https://CRAN.R‐project.org/package=emmeans

[ece310285-bib-0044] Llorens Climet, J. M. , & Martín Gil, Á. (2014). Guía de Gestión Integrada de Plagas Cítricos. Ministerio de Agricultura, Alimentación y Medio Ambiente.

[ece310285-bib-0045] Luff, M. L. (1965). The morphology and microclimate of *Dactylis Glomerata* tussocks. Journal of Ecology, 53(3), 771–787.

[ece310285-bib-0046] MAPA . (2022). Avance de datos de Cítricos, *Superficies y producciones anuales de cultivos*, Data file. https://www.mapa.gob.es

[ece310285-bib-0047] Marriott, C. A. , Bolton, G. R. , & Fisher, J. M. (2003). Changes in species composition of abandoned sown swards after imposing seasonal cutting treatments. Grass and Forage Science, 58(1), 37–49. 10.1046/j.1365-2494.2003.00350.x

[ece310285-bib-0048] Marshall, E. J. P. , & Moonen, A. C. (2002). Field margins in northern Europe: Their functions and interactions with agriculture. Agriculture, Ecosystems & Environment, 89(1–2), 5–21.

[ece310285-bib-0049] Merou, T. P. , Tsiftsis, S. , & Papanastasis, V. P. (2013). Disturbance and recovery in semi‐arid Mediterranean grasslands. Applied Vegetation Science, 16(3), 417–425. 10.1111/avsc.12013

[ece310285-bib-0050] Miettinen, A. , Korpela, E.‐L. , Hyytiäinen, K. , & Kuussaari, M. (2014). Cost‐effectiveness of agri‐environmental measures when aiming at promoting ecosystem service availability, species diversity or species of conservation concern. In *International Congress*, *August 26–29*, *2014*, Ljubljana, Slovenia.

[ece310285-bib-0051] Mockford, A. , Westbury, D. B. , Ashbrook, K. , Urbaneja, A. , & Tena, A. (2022). Structural heterogeneity of wildflower strips enhances fructose feeding in parasitoids. Agriculture, Ecosystems and Environment, 39, 108139. 10.1016/j.agee.2022.108139

[ece310285-bib-0052] Monzó, C. , Mockford, A. , Barreda, A. , & García, A. U. (2020). Cubiertas vegetales como estrategia de gestion de plagas en citricos. Agricultura: Revista Agropecuaria y Ganadera, 1037, 40–44.

[ece310285-bib-0053] Natural England . (2013). Entry level stewardship, environmental Stewardship handbook. Natural England.

[ece310285-bib-0054] Neuwirth, E. (2022). RColorBrewer: ColorBrewer palettes. https://CRAN.R‐project.org/package=RColorBrewer

[ece310285-bib-0055] Nowakowski, M. , & Pywell, R. (2016). Habitat creation and management for pollinators. Centre for Ecology & Hydrology. 10.1080/00016470052943982

[ece310285-bib-0056] Oliveira, G. , Nunes, A. , Clemente, A. , & Correia, O. (2012). Testing germination of species for hydroseeding degraded Mediterranean areas. Restoration Ecology, 20(5), 623–630. 10.1111/j.1526-100X.2011.00816.x

[ece310285-bib-0057] Parry, H. R. , Macfadyen, S. , Hopkinson, J. E. , Bianchi, F. J. J. A. , Zalucki, M. P. , Bourne, A. , & Schellhorn, N. A. (2015). Plant composition modulates arthropod pest and predator abundance: Evidence for culling exotics and planting natives. Basic and Applied Ecology, 16(6), 531–543. 10.1016/j.baae.2015.05.005

[ece310285-bib-0061] R Core Team . (2019). R: A language and environment for statistical computing. https://www.r‐project.org/

[ece310285-bib-0058] Ramírez, P. , & Lasheras, J. M. (2015). Guía de cubiertas vegetales en vid. https://www.juntadeandalucia.es/agriculturaypesca/ifapa/servifapa/registro‐servifapa/406aee3d‐31f0‐4f40‐8415‐9526cf178cf3

[ece310285-bib-0059] Roberts, H. A. , & Lockett, P. M. (1978). Seed dormancy and field emergence in *Solanum nigrum* L. Weed Research, 18(4), 231–241.

[ece310285-bib-0060] Rosa‐Schleich, J. , Loos, J. , Mußhoff, O. , & Tscharntke, T. (2019). Ecological‐economic trade‐offs of diversified farming systems – A review. In Ecological economics (pp. 251–263). Elsevier B.V. 10.1016/j.ecolecon.2019.03.002

[ece310285-bib-0062] RStudio Team . (2015). RStudio: Integrated development environment for R. http://www.rstudio.com/

[ece310285-bib-0063] Schutte, B. J. , Tomasek, B. J. , Davis, A. S. , Andersson, L. , Benoit, D. L. , Cirujeda, A. , Dekker, J. , Forcella, F. , Gonzalez‐Andujar, J. L. , Graziani, F. , Murdoch, A. J. , Neve, P. , Rasmussen, I. A. , Sera, B. , Salonen, J. , Tei, F. , Tørresen, K. S. , & Urbano, J. M. (2014). An investigation to enhance understanding of the stimulation of weed seedling emergence by soil disturbance. Weed Research, 54(1), 1–12. 10.1111/wre.12054

[ece310285-bib-0064] Shafie, F. A. , & Rennie, D. (2012). Consumer perceptions towards organic food. Procedia‐Social and Behavioral Sciences, 49, 360–367. 10.1016/j.sbspro.2012.07.034

[ece310285-bib-0065] Silva, E. B. , Franco, J. C. , Vasconcelos, T. , & Branco, M. (2010). Effect of ground cover vegetation on the abundance and diversity of beneficial arthropods in citrus orchards. Bulletin of Entomological Research, 100(4), 489–499. 10.1017/S0007485309990526 20102658

[ece310285-bib-0066] Simpson, G. L. (2019). permute: Functions for generating restricted permutations of data. https://cran.r‐project.org/package=permute

[ece310285-bib-0067] Souther, S. , Loeser, M. , Crews, T. E. , & Sisk, T. (2019). Complex response of vegetation to grazing suggests need for coordinated, landscape‐level approaches to grazing management. Global Ecology and Conservation, 20, e00770. 10.1016/j.gecco.2019.e00770

[ece310285-bib-0068] Stevenson, M. J. , Bullock, J. M. , & Ward, L. K. (1995). Re‐creating semi‐natural communities: Effect of sowing rate on establishment of calcareous grassland. Restoration Ecology, 3(4), 279–289.

[ece310285-bib-0069] Stewart, K. E. J. , Bourn, N. A. D. , & Thomas, J. A. (2001). An evaluation of three quick methods commonly used to assess sward height in ecology. Journal of Applied Ecology, 38(5), 1148–1154. 10.1046/j.1365-2664.2001.00658.x

[ece310285-bib-0070] Storkey, J. , & Westbury, D. B. (2007). Managing arable weeds for biodiversity. Pest Management Science, 63, 809–814. 17437252 10.1002/ps.1375

[ece310285-bib-0071] Sutter, L. , Albrecht, M. , & Jeanneret, P. (2018). Landscape greening and local creation of wildflower strips and hedgerows promote multiple ecosystem services. Journal of Applied Ecology, 55(2), 612–620. 10.1111/1365-2664.12977

[ece310285-bib-0072] Tena, A. , Pekas, A. , Cano, D. , Wäckers, F. L. , & Urbaneja, A. (2015). Sugar provisioning maximizes the biocontrol service of parasitoids. Journal of Applied Ecology, 52(3), 795–804. 10.1111/1365-2664.12426

[ece310285-bib-0073] Thomas, M. B. , Wratten, S. D. , & Sotherton, N. W. (1991). Creation of “Island” habitats in farmland to manipulate populations of beneficial arthropods: Predator densities and species composition. Journal of Applied Ecology, 28(3), 906–917. 10.1016/0006-3207(93)90399-L

[ece310285-bib-0074] Tilman, D. , Reich, P. B. , & Knops, J. M. H. (2006). Biodiversity and ecosystem stability in a decade‐long grassland experiment. Nature, 441(7093), 629–632. 10.1038/nature04742 16738658

[ece310285-bib-0075] Tuell, J. K. , Fiedler, A. K. , Landis, D. , & Isaacs, R. (2008). Visitation by wild and managed bees (Hymenoptera: Apoidea) to eastern U.S. native plants for use in conservation programs. Environmental Entomology, 37(3), 707–718. 18559176 10.1603/0046-225x(2008)37[707:vbwamb]2.0.co;2

[ece310285-bib-0076] Urbaneja, A. , Grout, T. G. , Gravena, S. , Wu, F. , Cen, Y. , & Stansly, P. A. (2020). Citrus pests in a global world. In M. Talon , M. Caruso , & F. G. Gmitter, Jr. (Eds.), The genus citrus. Elsevier.

[ece310285-bib-0077] van Rijn, P. C. J. J. , & Wäckers, F. L. (2016). Nectar accessibility determines fitness, flower choice and abundance of hoverflies that provide natural pest control. Journal of Applied Ecology, 53(3), 925–933. 10.1111/1365-2664.12605

[ece310285-bib-0078] Vickery, J. A. , Tallowin, J. R. , Feber, R. E. , Asteraki, E. J. , Atkinson, P. W. , Fuller, R. J. , & Brown, V. K. (2001). The management of lowland neutral grasslands in Britain: Effects of agricultural practices on birds and their food resources. Journal of Applied Ecology, 38(3), 647–664. 10.1046/j.1365-2664.2001.00626.x

[ece310285-bib-0079] Wäckers, F. L. , van Rijn, P. C. J. , & Bruin, J. (2005). Plant‐provided food for carnivorous insects: A protective mutualism and its applications. Cambridge University Press.

[ece310285-bib-0080] Wang, Y. , Naumann, U. , Wright, S. T. , & Warton, D. I. (2012). Mvabund‐ an R package for model‐based analysis of multivariate abundance data. Methods in Ecology and Evolution, 3(3), 471–474. 10.1111/j.2041-210X.2012.00190.x

[ece310285-bib-0081] Warton, D. I. , & Hui, F. K. C. (2011). The arcsine is asinine: The analysis of proportions in ecology. Ecology, 92(1), 3–10. 21560670 10.1890/10-0340.1

[ece310285-bib-0082] Warton, D. I. , Thibaut, L. , & Wang, Y. A. (2017). The PIT‐trap — A “model‐free” bootstrap procedure for inference about regression models with discrete, multivariate responses. PLoS One, 12(7), 1–18. 10.1371/journal.pone.0181790 PMC5524334 28738071

[ece310285-bib-0083] Westbury, D. B. , Woodcock, B. A. , Harris, S. J. , Brown, V. K. , & Potts, S. G. (2008). The effects of seed mix and management on the abundance of desirable and pernicious unsown species in arable buffer strip communities. Weed Research, 48(2), 113–123. 10.1111/j.1365-3180.2007.00614.x

[ece310285-bib-0084] Westbury, D. B. , Woodcock, B. A. , Harris, S. J. , Brown, V. K. , & Potts, S. G. (2017). Buffer strip management to deliver plant and invertebrate resources for farmland birds in agricultural landscapes. Agriculture, Ecosystems and Environment, 240, 215–223. 10.1016/j.agee.2017.02.031

[ece310285-bib-0085] Wickham, H. (2020). tidyr: Tidy messy data. https://cran.r‐project.org/package=tidyr

[ece310285-bib-0086] Wickham, H. , François, R. , Henry, L. , & Müller, K. (2020). dplyr: A grammar of data manipulation. https://cran.r‐project.org/package=dplyr

[ece310285-bib-0087] Woodcock, B. A. , Potts, S. G. , Tscheulin, T. , Pilgrim, E. , Ramsey, A. J. , Harrison‐Cripps, J. , Brown, V. K. , & Tallowin, J. R. (2009). Responses of invertebrate trophic level, feeding guild and body size to the management of improved grassland field margins. Journal of Applied Ecology, 46(4), 920–929. 10.1111/j.1365-2664.2009.01675.x

[ece310285-bib-0088] Woodcock, B. A. , Potts, S. G. , Westbury, D. B. , Ramsay, A. J. , Lambert, M. , Harris, S. J. , & Brown, V. K. (2007). The importance of sward architectural complexity in structuring predatory and phytophagous invertebrate assemblages. Ecological Entomology, 32(3), 302–311. 10.1111/j.1365-2311.2007.00869.x

[ece310285-bib-0089] Woodcock, B. A. , Westbury, D. B. , Potts, S. G. , Harris, S. J. , & Brown, V. K. (2005). Establishing field margins to promote beetle conservation in arable farms. Agriculture, Ecosystems and Environment, 107(2–3), 255–266. 10.1016/j.agee.2004.10.029

[ece310285-bib-0090] Yang, Y. Y. , & Kim, J. G. (2016). ‘The optimal balance between sexual and asexual reproduction in variable environments: A systematic review. Journal of Ecology and Environment, 40(1), 1–18. 10.1186/s41610-016-0013-0

